# What about the mothers? An analysis of maternal mortality and morbidity in perinatal health surveillance systems in Europe

**DOI:** 10.1111/j.1471-0528.2012.03330.x

**Published:** 2012-06

**Authors:** M-H Bouvier-Colle, AD Mohangoo, M Gissler, Z Novak-Antolic, C Vutuc, K Szamotulska, J Zeitlin, for The Euro-Peristat Scientific Committee

**Affiliations:** a Institut National de la Santé et de la Recherche médicale-Unité 953 Recherche épidémiologique en santé périnatale et santé des femmes et des enfants—INSERM, UMR S953 Epidemiological Research Unit on Perinatal Health and Women's and Children's Health, UPMC University ParisParis, France; b Netherlands Organization for Applied Scientific Research TNOLeiden, the Netherlands; c National Institute for Health and Welfare, Helsinki, Finland and Nordic School of Public HealthGothenburg, Sweden; d Department of Obstetrics and Gynaecology, Division of Perinatology, University Medical CentreLjubljana, Slovenia; e Leiter der Abteilung für Epidemiologie Zentrum für Public Health der Med. Univ. WienAustria; f Department of Epidemiology National Research Institute of Mother and ChildWarsaw, Poland

**Keywords:** European Union, hospital data, maternal mortality ratio, severe obstetric complications

## Abstract

**Objective:**

To assess capacity to develop routine monitoring of maternal health in the European Union using indicators of maternal mortality and severe morbidity.

**Design:**

Analysis of aggregate data from routine statistical systems compiled by the EURO-PERISTAT project and comparison with data from national enquiries.

**Setting:**

Twenty-five countries in the European Union and Norway.

**Population:**

Women giving birth in participating countries in 2003 and 2004.

**Methods:**

Application of a common collection of data by selecting specific International Classification of Disease codes from the ‘Pregnancy, childbirth and the puerperium’ chapter. External validity was assessed by reviewing the results of national confidential enquiries and linkage studies.

**Main outcome measures:**

Maternal mortality ratio, with distribution of specific obstetric causes, and severe acute maternal morbidity, which included: eclampsia, surgery and blood transfusion for obstetric haemorrhage, and intensive-care unit admission.

**Results:**

In 22 countries that provided data, the maternal mortality ratio was 6.3 per 100 000 live births overall and ranged from 0 to 29.6. Under-ascertainment was evident from comparisons with studies that use enhanced identification of deaths. Furthermore, routine cause of death registration systems in countries with specific systems for audit reported higher maternal mortality ratio than those in countries without audits. For severe acute maternal morbidity, 16 countries provided data about at least one category of morbidity, and only three provided data for all categories. Reported values ranged widely (from 0.2 to 1.6 women with eclampsia per 1000 women giving birth and from 0.2 to 1.0 hysterectomies per 1000 women).

**Conclusions:**

Currently available data on maternal mortality and morbidity are insufficient for monitoring trends over time in Europe and for comparison between countries. Confidential enquiries into maternal deaths are recommended.

## Introduction

During the 1970s and the 1980s, maternal and child health policies focused more attention on infants than on mothers. For instance, antenatal care has focused more on the prevention of health problems for the fetus or infant rather than on the organisation of obstetric and intensive care for the mothers in case of severe maternal complications. At the end of the 1980s, maternal mortality was labelled ‘a neglected tragedy’.^1^,^2^ In 1987, a plea for safe motherhood worldwide was launched. This led to a greater international focus on maternal health because of high maternal mortality ratios in low-income countries. In Europe, as in other high-income countries, specific surveys were carried out on maternal mortality and initiatives for severe maternal morbidity were taken.^3^–^7^ An important question is therefore the extent to which these initiatives have improved the capacity to analyse and to develop public-health strategies for maternal health in Europe.

Five million women give birth each year in the European Union. Another million women have failed pregnancies with first-trimester losses. Overall in the European Union, between 335 and 1000 women are estimated to die during or because of pregnancy, delivery or the puerperium.^8^ The EURO-PERISTAT group, a European collaboration established to develop an information system on perinatal health in Europe, recommends the maternal mortality ratio (MMR) and the causes of maternal death as two principal indicators of maternal health.^5^ Maternal mortality is considered to be an important indicator of health system performance^4^ even in high-income countries where maternal deaths are very rare but are considered sentinel events that raise questions about the effectiveness and quality of care. Vital registration and healthcare information systems exist in all member states, and provide an opportunity to produce direct estimates of MMR using a common classification of causes of death.

In addition to maternal mortality, EURO-PERISTAT recommends an indicator of severe maternal morbidity. The difficulties involved in establishing common definitions of maternal morbidity have been apparent for some time. In the 1990s, the European Concerted Action on Maternal Mortality and Severe Morbidity (the MOMS study) with collaborators from 15 countries studied three types of severe maternal morbidity complications (severe postpartum haemorrhage, eclampsia/pre-eclampsia and sepsis) for which the participants drew up common definitions.^6^ Special epidemiological surveys were carried out in the participating countries to estimate the prevalence of these maternal morbidities. These showed wide differences between the morbidity rates. The study concluded that these were probably the result of differences in the data survey procedures (prospective or retrospective for example). After a review of the literature and based on studies performed in Europe, the EURO-PERISTAT group proposed in 2004 a series of severe maternal conditions linked to pregnancy and childbirth, which might be generated using data from routine systems (hospital discharge registers and medical birth registers).

This article reports on the results of data collection on maternal health indicators (mortality and morbidity) in 25 European Union member states and Norway to produce the European Perinatal Health Report. Our aim was to analyse current capacity to monitor trends and differences in maternal mortality and morbidity in Europe and to compare the MMR with information from other sources—notably confidential enquiries.

## Methods

We used data from the EURO-PERISTAT project, the methods for which are described below and elsewhere,^8^,^9^ as well as data from published reports of national enquiries or specific studies into maternal deaths.

### Definitions

#### Maternal mortality

Internationally accepted definitions for indicators of maternal mortality and obstetric causes of death have been published by the World Health Organization.^10^ Maternal death is defined as the death of a woman while pregnant or within 42 days of the termination of pregnancy irrespective of the duration and site of the pregnancy for any cause related to or aggravated by the pregnancy or its management, but not from accidental or incidental causes. Maternal deaths are subdivided into direct and indirect obstetric causes of death (Chapter O, digits from O 00 to O 99) of the 10th revision of the International Classification of Diseases (ICD-10), which defines the classification of pathologies related to pregnancy, childbirth and the puerperium. Currently all national cause-of-death registries in the study countries record deaths coded according to ICD-10.

For the EURO-PERISTAT project we compiled data about all maternal deaths and deaths attributed to the main causes: ectopic pregnancy or abortion, complications of hypertension, antepartum and postpartum haemorrhage (PPH), uterine rupture, amniotic fluid embolism or thromboembolism, sepsis or chorioamnionitis, other direct obstetric causes, indirect obstetric causes and unknown causes. These causes were then aggregated into nine major categories, haemorrhages and uterine ruptures, amniotic fluid embolism, thromboembolism, complications of hypertension, ectopic pregnancies and abortions, anaesthetic complications, other direct obstetric causes, indirect causes, and unknown causes.^4^

As a consequence of the very small number of deaths each year in most countries, we requested data covering at least 2 years (2003 and 2004). Small countries provided data for longer periods to provide a more reliable estimate for their MMR. For example, Luxembourg provided data for 5 years. Two large countries, Germany and Italy, provided data for only 1 year.

The MMR is defined as the number of all maternal deaths from direct and indirect obstetric causes per 100 000 live births. We did not calculate MMRs by cause of death because of the small number of deaths.

We calculated the percentage of deaths in each group of causes, as the number of the deaths reported (numerator) by the total number of maternal deaths (denominator).

#### Severe acute maternal morbidity

The EURO-PERISTAT working group conducted an extensive review of potential maternal morbidity indicators. We identified four possible sources for morbidity data used in the scientific literature: (i) hospital discharge registers and databases, (ii) financial data about hospital care, (iii) obstetric quality registers, and (iv) medical birth registers and databases. Because the most frequently used sources of data are hospital discharge registers and databases, we selected indicators that could be generated using data usually included in these databases. Our hypothesis was that all women with severe acute maternal morbidity (SAMM) would receive hospital care and so be included in hospital databases.

The EURO-PERISTAT maternal morbidity indicators included both management-based criteria and clinical diagnoses. The choice of indicators was agreed upon in a meeting of the EURO-PERISTAT committee, based on results from EURO-PERISTAT I^5^ and our literature review. Four indicators, had been suggested in the first phase of the project (eclampsia, surgery, blood transfusion, and intensive-care unit admission). Embolisation was added as a fifth indicator. Our initial intent was to present each indicator separately and also to combine them in a composite indicator of SAMM. Appendix S2 (see Supporting Information) gives the exact definitions for each indicator. The indicators were defined as the number of women experiencing the condition or procedure as a rate (per 1000) of all women giving birth to one or more live or stillborn babies.

##### Commentary on ‘What about the mothers? An analysis of maternal mortality and morbidity in perinatal health surveillance systems in Europe’

This important paper from EURO-PERISTAT concludes correctly that routine registration data on maternal mortality are insufficient to monitor trends over time and for comparing countries. Unfortunately the World Health Organization relies solely on such data when producing maternal mortality estimates worldwide (Hogan et al., *Lancet* 2010;375:1609–23). More sophisticated systems such as confidential enquiries reveal substantial under-reporting, with rates of almost 20% in France, 33% in the Netherlands, 38% in Austria, almost 50% in the UK, 50% in Poland, 75% in Italy and 87% in Slovenia. This makes routinely collected vital statistics less useful and comparison between countries meaningless. Sweden will soon publish data from active surveillance, which will illuminate the extremely low official maternal mortality ratio in that country.

Under-reporting has several causes, including incomplete ascertainment or misclassifications of maternal deaths as non-maternal. When immigrant deaths are not included (as in Austria) or indirect maternal deaths are left out (as in Norway), comparison between different countries becomes meaningless, especially as we know that immigrants are often disproportionally represented and indirect deaths comprise a high proportion. In this study, five countries reported zero indirect deaths. That may be understandable when the total numbers are low (Luxembourg, four; Lithuania, six and Estonia, eight). It is, however, likely that Spain with 41 and Poland with 31 deaths, did not (like Norway) report their indirect deaths.

Substantial differences within countries exist, with large variations between regions, cities, provinces and even neighbourhoods (Saucedo et al., *BJOG* 2012;119:573–81; de Graaf et al., *BJOG* 2012;119:582–8). Higher maternal mortality ratios in deprived areas (as detected by postal codes) point to the socio-economic and multi-ethnic determinants of health, indicating that serious health inequalities still exist within our welfare states.

The relatively short period of data collection leading to few maternal deaths being recorded, e.g. Luxembourg (*n* = 2 for 2000–04), Slovenia, Sweden and Norway (*n* = 4), Greece (*n* = 2) is a limitation.

Causes of maternal deaths vary substantially between countries, although obstetric haemorrhage remains the most frequent cause. Deaths from haemorrhage range between 0 and 50% of all maternal deaths, and those from amniotic fluid embolism are between 0 and 20%. Amniotic fluid embolism often involves serious haemorrhage, so classifications are bound to overlap. As maternal deaths tend to become litigation cases, clinicians sometimes may prefer the ‘less avoidable’ cause of amniotic fluid embolism to obstetric haemorrhage. Unknown causes differed between 0 and 47% of the deaths. In ten of the 16 countries no maternal deaths of ‘unknown origin’ were reported. This gives rise to doubts about the figures, because documentation is often a problem, even in a confidential enquiry. Moreover, maternal deaths are often complex. Recently, when 25 experts from the International Network of Obstetric Survey Systems (INOSS) at their second annual INOSS meeting in Leiden tried to classify cases of maternal mortality from France and the Netherlands, consensus could not easily be reached, and an underlying cause could not always be assigned. Such differences can only be resolved by in-depth audit of every case of maternal death, with prospective data collection. This can be achieved when countries have national enquiries in place, and that is the most important recommendation from this study.

**J van Roosmalen**

Department of Obstetrics, Leiden University Medical Centre, the Netherlands

### Data collection

Members of the EURO-PERISTAT Scientific Committee were responsible for organising data collection in their own country. They either compiled the data themselves from data published by national organisations or provided the names of people to whom the data collection instruments should be sent. The aim of EURO-PERISTAT was to gather population-based data at a national level. If these were not available, regional data were accepted if they covered a geographically defined population. Only data from existing routine data sources—including vital registration systems, hospital administrative data, systems or regular surveys—were used. The Scientific Committee member for each participating country was responsible for selecting the most appropriate data source. Appendix S1 (see Supporting Information) gives the data source used in each country.

Aggregated data were collected using an excel-based system. We asked for data for 2004 or the latest available year before 2004, except for maternal mortality for which data for two or more years were requested. TNO Quality of Life in the Netherlands was responsible for developing the data collection instrument and overseeing the collection process.

The second source of data was publications of national results from countries that studied maternal deaths using enhanced systems of registration by confidential enquiries into maternal deaths and/or data linkage. We drew on published reports from Austria,^11^ Denmark,^12^ Finland,^13^ France,^14^ Italy (five regions),^15^ the Netherlands,^16^ Norway,^17^ Slovenia^18^ and the UK.^19^ These sources are listed in Appendix S1 (see Supporting Information). We also included comparison data from a recent international study using routinely collected medical causes of death data, which were corrected for under-reporting.^20^ Data from these studies were used for external validation of national MMR. In addition, for those countries that carry out routine surveillance of maternal deaths using enhanced systems (Finland, France, the Netherlands, Slovenia and the UK), we compared national MMR with MMR from other countries with similar infant mortality rates, but no enhanced system for ascertaining maternal deaths.

## Results

### Maternal mortality ratios

All countries contributed data on maternal deaths except Cyprus, Ireland and Slovakia. Belgium, Denmark, Greece, Norway, Portugal and Sweden provided only the total number of deaths without information about their causes. The total number of maternal deaths reported by country and by year varied from 0 in Malta, in both years and in Norway and Slovenia in 2004, to 55 in both France and the UK in 2003.

The total number of deaths per country ranged from 0 to108; the total number of live births ranged from <8000 to over 1.5 million. The highest ratio was reported in Estonia, with 29.6 per 100 000 live births and the lowest was 0 in Malta, as shown in Table 1. Austria, Belgium, France and Hungary had rates around the EU average (of 6.2 per 100 000 live births). Sweden and Greece reported low ratios of 2.0 per 100 000.

**Table 1:** Live births, maternal deaths, maternal mortality ratios, repartition (%) of the maternal deaths according to the obstetric causes, by country in 2003/04

Countries*	Live births (*n*)	Maternal deaths (*n*)	MMR per 100 000 live births	Causes of maternal deaths (%)**						
				
				H	AFE	OTE	CHT	OD	AI	UK
Austria^1^	155 912	10	6.4	0	10	10	20	10	50	0
Belgium^2^	156 167	7	4.5							
Cyprus	No data									
Czech Republic	191 349	19	9.9	11	16	21	0	26	21	5
Denmark^2^	129 466	12	9.3							
Estonia^3^	27 028	8	29.6	0	20	0	20	60	0	0
Finland	114 018	9	7.9	11	11	0	11	44	22	0
France	1 529 280	107	7.0	18	14	14	14	28	8	4
Germany^4^	1 320 820	67	5.1	7	5	7	2	16	16	47^3^
Greece^5,2^	104 355	2	1.9							
Hungary	190 274	14	7.4	14	0	14	0	36	29	7
Ireland	No data									
Italy^4,6^	539 066	17	3.2	18	6	6	6	53	6	6^3^
Latvia	41 340	5	12.1	0	20	20	0	0	60	0
Lithuania	61 017	6	9.8	0	0	17	17	67	0	0
Luxembourg^7^	27 252	2	7.3	0	0	0	0	100	0	0
Malta	7923	0	0.0							
The Netherlands	362 012	32	8.8	9	0	13	13	13	34	19
Norway^2^	113 409	4	3.5							
Poland	707 203	31	4.4	39	13	3	6	39	0	0
Portugal^2^	221 945	17	7.7							
Slovakia	No data									
Slovenia^7^	34 907	4	11.5	50	0	25	0	0	25	0
Spain	896 472	41	4.6	0	0	0	25	75	0	0
Sweden^2^	200 316	4	2.0							
United Kingdom	1 411 545	108	7.7	6	14	8	9	41	22	0
All countries	8 308 853	519	6.2	13	11	10	9	32	17	8
Countries with <10% of unknown causes	6 626 021	420	6.3							

AFE, amniotic fluid embolism; AI, all indirect; CHT, complications of hypertension; H, obstetric haemorrhage; OD, other direct (other direct obstetric causes: chorioamnionitis/sepsis, abortion/ectopic pregnancy; anaesthetic; uterine rupture and others); OTE, Other thromboembolisms; UK, unknown.

* :^1^In Austria indirect causes of maternal death are registered since 2004. ^2^Belgium, Denmark, Greece, Norway, Portugal and Sweden provided no data on maternal mortality by cause of death. ^3^Estonia provided data for the years 2004 and 2005, and Slovenia provided data for the years 2001 and 2002. ^4^Germany and Italy provided data on maternal mortality by cause of death for 1 year only, respectively 2004 and 2002. ^5^Greece provided data for 1 year, 2003. ^6^Italy provided data on maternal mortality by cause of death based on ICD-9 codes. ^7^Luxembourg provided data on maternal death for the years 2000–2004 and Slovenia for the years 2001 and 2002.

** We are very concerned by the fact that calculating percentages with so small numbers sometimes is not pertinent, but we need to have the same presentation for all the members of the EU.

### Causes of maternal deaths

The profile of causes varied substantially from country to country (Table 1) because of differences in the proportions of deaths attributed to unknown causes: seven countries did not use this category at all, whereas in other countries many deaths had no cause stated. This problem was greatest in the Netherlands and in Germany where, respectively, 19 and 47% had no reported cause. Nevertheless, the general European profile of stated direct obstetric causes of death shows that all obstetric haemorrhages, including a majority of PPH by atony, accounted for the highest proportion of maternal deaths in participating countries (13%). In countries that reported it as a direct cause of maternal death, the proportion of haemorrhages ranged from 7% in Germany to 67% in Slovenia. Three other direct causes each accounted for around 9–11% of maternal deaths: thromboembolisms (10% in the European Union, ranging from 3% in Poland to 33% in Slovenia), complications of hypertension (9% in the European Union, ranging from 2% in Germany to 25% in Spain), and amniotic fluid embolism (11% in the European Union, ranging from 5% in Germany to 20% in Latvia and Estonia).

In nine countries that provided data to the EURO-PERISTAT project, we were able to check the completeness for routinely collected data about maternal deaths by comparing ratios with other published studies on maternal deaths: in Austria, Denmark, Finland, France, Italy, the Netherlands, Norway, Slovenia and the UK. Table 2 shows that the official MMRs under-ascertained maternal mortality in all the European countries where it was possible to make comparisons. For Norway and Denmark, the confidential enquiries were performed at earlier dates and cannot be compared with the EURO-PERISTAT data. Slovenia published a first report on its systematic audit from national registries resulting in an MMR of 9.8 per 100 000 for the period 1985–94. This is equal to the rate included in the EURO-PERISTAT report, but covers a longer period. The under-ascertainment was generally between 20 and 50%. Often, underestimation was higher in countries with a lower official rate. We also compared the official MMRs in countries that routinely monitor maternal deaths using audits or linkages or confidential enquiries (Finland, France, the Netherlands, Slovenia and the UK) with the official MMR in countries that do not, but that have a similar level of infant mortality (Austria, Belgium, Czech Republic, Denmark, Germany, Italy, Luxembourg, Poland, Portugal, Spain, Sweden). In all five countries with specific audits, linkage or enquiries, MMRs were at least seven per 100 000 live births, whereas seven of the 11 other countries (63%), had MMRs <7.

**Table 2:** Maternal mortality data according to different sources, numbers, ratios per 100 000 live births and percentage of underestimation, in France, Finland, Italy, Netherlands and UK around 2000–04

Countries Years	(*a*) Confidential enquiries *n* maternal deaths	(*b*) Civil registration causes of death	(*c*) Under-estimation* (%)	MMRs according to different sources		
				
				Confidential enquiries	Vital data (last period)	Hogan** estimates 2000
**Austria**						
1980–98	191	119	38.0	NA	NA	5 (4–7)
2003/04***		10			6.4	
**Denmark**						
1985–94	60			9.8		7 (5–9)
2003/04		12			9.3	
**Finland**						
1999	6	3	50.0	5.3	2.6	7 (5–9)
2003/04		9			7.9	
**France**						
1999	58	47	19.0	NA	7.4	11 (10–13)
2001–06	463	384	17.1	9.6	8.0	
2003/04		107			7.0	
**Italy**						
2000–07	118	NA	74.6	11.8	NA	5 (4–6)
2003/04		17			3.2	
**The Netherlands**						
1993–2005	309	208	32.7	12.1	8.1	8 (10–11)
2003/04		32			8.8	
**Norway**						
1976–95	61****			5.5**		7 (5–10)
2003/04		4			3.5	
**Slovenia**						
2003–05	8*****	1	87.5	9.4	1.9	21 (15–29)
2003/04		4			11.5	
**United Kingdom**						
2000–02	261	136	47.9	13.1	6.8	
2003–05	295	149	49.5	13.9	7.0	8 (7–10)
2003/04		108			7.7	

Sources, EURO-PERISTAT, Confidential enquiries and specific surveys.

* Underestimation : *c* = (*a*) − (*b*)/(*a*) × 100, except for Austria.

** Hogan estimations are based on modelling of vital data.

*** All 2003/04 data are taken from EURO-PERISTAT.

**** Norway recorded direct obstetric causes of deaths only.

***** Slovenia among the eight deaths, three were late maternal deaths (≥42 days).

### Maternal morbidity

Sixteen member states provided data for at least one of the indicators of SAMM but only three provided data about all the categories, including admission into intensive-care units (Table 3).

**Table 3:** Severe maternal morbidity rates per 1000 women by pathologies or interventions, according to the countries

Countries	No. of women	Eclampsia	ICU admission	Blood transfusion whatever the number of units*	Hysterectomy	Embolisation
Austria**						
Belgium Flanders	59 956	NA	NA	11.5	NA	NA
Cyprus**						
Czech Republic	96 771	0.2	NA	NA	0.8	NA
Denmark	63 781	0.3	NA	11.0	0.3	0.0
Estonia	13 879	0.6	NA	NA	0.9	NA
Finland	56 878	0.2	NA	0.1	0.2	0.2
France	774 870	1.1	0.5	2.1	0.3	0.3
Germany–Bavaria	105 490	0.7	3.1	10.7	1.0	0.0
**Greece**						
Hungary	93 913	0.5	NA	NA	1.0	0.0
Ireland**						
Italy	534 568	1.6	NA	4.6	0.9	0.0
Latvia	20 256	0.4	NA		0.8	NA
Lithuania**						
**Luxembourg**						
Malta	3838	1.3	NA	5.2	0.5	NA
The Netherlands	187 910	0.7	2.2	6.4	0.3	0.3
Norway**						
Poland	213 190	0.2	NA	NA	NA	NA
Portugal**						
Scotland only	53 342	0.6	NA	NA	0.2	NA
Slovakia**						
Slovenia	17 629	1.1	NA	10.6	0.6	NA
Spain–Valencia	38 389	0.3	NA	6.5	0.3	NA
Sweden**						
United Kingdom (Wales and Scotland)	82 911	0.67***	NA	NA	0.13***	NA

* The number of transfusion units provided by the countries were so heterogeneous (three units or more, five units or more, other amount, no units specified) that we summarised the data in only one category.

** No data provided; Norway did not participate in the data collection.

*** The rates were estimated from two nations only, Wales and Scotland.

Hysterectomy for PPH and eclampsia were the two complications for which data were most often available. There was a five-fold country difference in the estimates of incidence, but the range was moderate at 0.2–1.0 per 1000 women giving birth, as was the range for eclampsia: 0.2–1.6 per 1000. In contrast, data on intensive-care unit admission were not generally available and the between-country differences were large (six-fold); There were very wide variations in blood transfusion data, most probably because of differences in inclusion criteria. Data on embolisation of uterine arteries were available for only seven countries and the rates varied between 0.0 and 0.3 per 1000.

## Discussion

This attempt to gather data on SAMM and maternal mortality at a European level using routine national systems show that currently available data are insufficient. Fortunately, some countries have data from enhanced systems for identification of maternal death and these make it possible to quantify the shortfalls in data from national statistical offices. This is a crucial issue because the absence of good data on maternal mortality and morbidity undermines national and European capacity to monitor maternal health in Europe, and to permit comparisons between countries or surveillance of trends over time. Our results suggest that calls for a greater focus on mothers are still highly relevant for European countries and may also be for other high-income countries, as recently affirmed in the USA.^7^

For the measure of maternal mortality, there are two problems, completeness of ascertainment and quality of coding. The comparison of data from routine cause of death certificates with enhanced systems for studying maternal deaths identified substantial underestimation of between 20 and 50% in MMRs. Furthermore, countries with continuous audits had higher reported MMR than countries that have not implemented these initiatives, probably because of increased awareness of maternal deaths, which improves the routine reporting of these deaths. From this perspective, the very low mortality ratios in some countries are highly suggestive of a failure to adequately count maternal deaths. It would be interesting to study why these systems are missing maternal deaths and in particular, whether this is associated with the completeness of ascertainment or the procedures for coding. The absence of deaths from indirect obstetric causes in some countries, for example Poland or Spain, might be the result of under-ascertainment, or coding procedures, or both. A further issue that affects completeness of ascertainment and comparability is whether migrant or foreign citizens are included in official mortality statistics: for example, in France they are, but in Austria they are not. Migrant women have been found to have higher MMR than non-migrant women in the countries to which they migrate.^21^,^22^

These routinely reported data are those regularly used at the international level instead of the results from enhanced systems of maternal death recording. The recent paper by Hogan et al.,^20^ an attempt at the international level to rectify the well-known under-ascertainment of MMRs by using models based on vital statistics data in industrialised countries, was based on national statistical office data. Consequently, their estimates were consistently below the true values in the countries where alternative data sources were available. Nevertheless, in other countries, such as Greece, Malta and Sweden, their corrections led to values that were two or three times higher than ratios reported in national statistics.

There are validated methods for improving statistics on maternal mortality, including setting up systems of enquiries. Adding a pregnancy check box to the national medical death certificate is a first step, as shown recently in a study from the USA,^23^ but this is not sufficient, as witnessed by continued under-reporting in countries, such as France since 2000 and others where pregnancy check boxes have been implemented. To insure the completeness of registration, more comprehensive solutions involve data linkage of cause of death registers with medical birth registers or birth registration records (Denmark, England and Wales, Finland, Norway and Slovenia), abortion records (Finland) and hospital records (Denmark and Finland). National systems of confidential enquiries into maternal deaths exist in France, the Netherlands, Slovenia and the UK and these also rely in part on linkage systems for identifying cases.^14^,^16^,^18^,^19^ Implementing data linkage and especially confidential enquiries in all European countries would substantially improve the ascertainment of maternal death.

There are also major differences between countries in reporting causes of death. This problem is more difficult to resolve because of the small number of deaths and the complexity of the multiple complications that lead to a maternal death. Many women die in an intensive-care unit, often days or weeks after delivery, and the certifying physician may not always be aware of the details of the pregnancy. A previous European study showed that the differences in coding the underlying cause of death by the national statistical offices can lead to underestimation or overestimation of the MMR compared with standardised coding by a European panel of experts;^24^ these discrepancies were confirmed in a subsequent study comparing Europe with the USA.^13^,^25^

Another important issue that limits the use of maternal mortality indicators for surveillance of maternal health in pregnancy is the small number of deaths. For example, the relatively high MMR for Estonia is based on only eight maternal deaths; On the other hand, one maternal death in Malta would have increased its MMR from 0.0 to 12.6 per 100 000. Given this degree of variation, we would recommend that future international data collection and reporting be based on averages over 5 years instead of 2 years to reduce the effects of variations in the MMRs caused by the small number of maternal deaths, in medium-sized as well as small countries. This issue highlights the importance of developing valid indicators of severe maternal morbidity which have a higher incidence and therefore have a greater potential to measure trends in maternal health over time.

Our results show, however, that data on maternal morbidity are scarce and their quality is inadequate. We had expected that the incidence of embolism, eclampsia, blood transfusion and surgery for PPH would be readily derived from the data files compiled at hospital level. We know that the majority of the European countries have hospital discharge registers or databases that are used to monitor hospital activity and to allocate resources to hospitals. As the morbidity outcomes and procedures we chose take place in hospital at or shortly after delivery, the cases should be included in these systems. Many countries were not able to report the number of women; these data may exist, but they were not currently available for this purpose. The EURO-PERISTAT project compiled data that are currently used to evaluate perinatal health outcomes.

A future line of research would be to request data on hospital stays associated with childbirth from hospital discharge databases and validate the accuracy of reporting of some specific and crucial diagnoses for which there are commonly accepted definitions, including eclampsia, thromboembolism and sepsis. Results from new national and international initiatives should be taken into consideration when refining these definitions of severe maternal morbidity.^26^–^28^

Retrospective studies have been conducted in the USA, Canada and Australia on hospital databases or discharge summaries, according to a definition of SAMM that combines codes of procedures and diagnoses and that therefore depends on the available information.^29^–^31^ This type of study has the advantage of feasibility, immediately available data and a large sample size. Limitations include use of codes from the ICD of the World Health Organization to identify SAMM events; although the ICD has a section on direct and indirect maternal complications, it does not define their severity. There is also significant variation in the reporting of diagnoses that are not the main diagnoses. Moreover, hospital data are not able to distinguish morbidity associated with pregnancy (temporal association) from maternal morbidity directly caused by pregnancy (temporal and causal association). In some systems also there is a risk of counting the same woman in the same pregnancy several times unless there is a unique identifier and admissions are linked. The validity and accuracy of the reported conditions in the hospital data may be not checked^32^ in a detailed manner because of the large numbers of records involved.

Using hospital discharge data would also make it possible to record admission to intensive-care units, to which we are strongly favourable. Even though intensive-care admission depends in part on the way health care is organised, it can mark a critical event, a so called ‘near miss’.^33^ This identifies a situation in which resuscitation by an intensive-care specialist was required, as confirmed by recent studies.^34^,^35^ Since 2006, the intensive-care national audit and research centre (ICNARC) has analysed admissions of women of reproductive age among the admissions to adult general intensive-care units, in England and Wales and Northern Ireland.^36^ The LEMMoN Study in the Netherlands included a chapter about obstetric intensive-care unit admissions.^28^ In France, these admissions are well recorded in hospital discharge data.^32^

Epidemiological studies focusing on specific aspects of SAMM are more informative and provide an essential complement to routine reporting and some are already under way. They are usually population-based surveys giving better estimates of the severe maternal morbidity rates. The Scottish confidential audit of severe maternal morbidity is the oldest survey since 2003^26^ and allows calculation of SAMM indicators annually. The United Kingdom Obstetrical Surveillance System (UKOSS) covering the UK^36^ is an ongoing system that focuses in turn on specific types of rare severe maternal morbidity. Other studies, such as the LEMMoN^28^ study in the Netherlands, the recently started Nordic project (NOSS) or the French Severe maternal morbidity: measurement, determinants and quality of care project (EPIMOMS), are prospective but transversal approaches limited to a specific time period. Neither the UK study nor the others are designed to enable routine follow up over time.

## Conclusion and recommendations

Despite the existence of longstanding cause of death registration systems and hospital morbidity registers in European countries, currently available data for surveillance of maternal morbidity and mortality associated with pregnancy, childbirth and the postpartum period are inadequate. All countries should be encouraged to use validated methods to improve the ascertainment of maternal deaths and in particular confidential enquiries and data linkage. Better use of data available in hospital discharge databases should make it possible to identify indicators of morbidity that can be validly compared.

## Appendix

### The Euro-Peristat Scientific Committee

Christian Vutuc (Austria), Sophie Alexander (Belgium), Pavlos Pavlou (Cyprus), Petr Velebil (Czech Republic), Jens Langhoff Roos (Denmark), Luule Sakkeus (Estonia), Mika Gissler (Finland), Béatrice Blondel (France), Nicholas Lack (Germany), Aris Antlaklis (Greece), István Berbik (Hungary), Sheelagh Bonham (Ireland), Marina Cuttini (Italy), Jautrite Karaskevica (Latvia), Jone Jaselioniene (Lithuania), Yolande Wagener (Luxembourg), Miriam Gatt (Malta), Jan Nijhuis (The Netherlands), Lorentz Irgens (Norway), Katarzyna Szamotulska (Poland), Henrique Barros (Portugal), Mária Chmelová (Slovakia), Živa Novak-Antolic (Slovenia), Francisco Bolúmar (Spain), Gunilla Lindmark (Sweden), Alison Macfarlane (United Kingdom).

## Supporting Information

**Appendix S1.** Data sources used for maternal mortality and morbidity statistics in the EURO-PERISTAT project.

**Appendix S2.** Definitions used for maternal morbidity indicators.
