# Supplementary material for: What about the mothers? An analysis of maternal mortality and morbidity in perinatal health surveillance systems in Europe
Source: BJOG. 2012 Jun;119(7):880–90. doi: 10.1111/j.1471-0528.2012.03330.x (PMC3472023; doi:10.1111/j.1471-0528.2012.03330.x)
Supplement: Supplementary file 1 [file bjo0119-0880-SD1.pdf]

Appendix S1: Data sources used for maternal mortality and morbidity statistics in the EURO-PERISTAT project

Countries	**Sources for maternal mortality data**		**Data source for maternal morbidity**
	Routinely collected data	Confidential enquiries or enhanced systems of collection	
Austria	Cause of death statistics	Statistics Austria and Confidential enquiries to Departments of Gynaecology and Obstetrics and the Institutes of Pathology and Forensic medicine	--
Belgium	Flanders + Brussels vital records		SPE – birth register
Czech Republic	Czech Statistical Office, death certificates		Database of aggregated data of the Czech Society of Perinatal Medicine
Denmark	National register No data by cause	National Medical Registers Death certificates	Danish perinatal database
Estonia	Statistics Estonia(death registry for causes of deaths) + Estonian medical birth registry		Estonian medical birth registry
Finland	Cause-of-Death Register	Cause of Death Register, Medical Birth Register, Hospital Discharge Register, Abortion Register	Hospital Discharge Register
France	National statistics of causes of death	National Expert Committee on Maternal Mortality	PMSI – Hospital Discharge Data
Germany	www.bqs-online.de (Quality register) & www.destatis.de (National statistics)		Hospital quality birth register www.bqs-online.de
Greece	--		--
Hungary	National Institute of Obstetrics and Gynecology		National Institution of Obstetrics and Gynecology
Ireland	--		--
Italy	National Register of Deaths - Istat National Institute of Statistics	National Institute of Health and five collaborative regions	SDO (Scheda di Dimissione Ospedaliera, or Hospital Discharge Form)
Latvia	Mortality statistics / Ministry of health		Newborns Register of Latvia
Lithuania	Database of the Demographic Statistics, death certificates		--
Luxembourg	Mortality statistics / Ministry of health		--
Malta	National Mortality Register		National Obstetrics Information System (NOIS)
The Netherlands	Audit Committee on maternal mortality	Dutch Maternal Mortality Committee	LEMMoN Study
Norway	Medical birth registry of Norway	Cause of Death Registry, Statistics Norway and the Medical Birth Registry of Norway	Medical birth registry of Norway
Poland	Central Statistic Office		Hospital discharge data
Portugal	INE - Estatísticas Demográficas		--

Appendix S1. *(continued)*

Countries	Sources for maternal mortality data		Data source for maternal morbidity
	Routinely collected data	Confidential enquiries or enhanced systems of collection	
Slovakia	--		--
Slovenia	Mortality database, The Institute of Public Health of the Republic of Slovenia	2003-2005: National Perinatal Information System, Information system on Fetal death, Mortality Data base	National perinatal system of Slovenia
Spain	National Institute for Statistics. Mortality Register		CMBD (Hospital Registers including private hospitals)
Sweden	Cause of death register		--
United Kingdom	Civil registration of births and deaths for England and Wales, Scotland, Northern Ireland	Centre for Maternal and Child Health Enquiries	Scottish Morbidity Record SMR and Patient Episode Data Wales
