# Supplementary material for: What about the mothers? An analysis of maternal mortality and morbidity in perinatal health surveillance systems in Europe
Source: BJOG. 2012 Jun;119(7):880–90. doi: 10.1111/j.1471-0528.2012.03330.x (PMC3472023; doi:10.1111/j.1471-0528.2012.03330.x)
Supplement: Supplementary file 2 [file bjo0119-0880-SD2.pdf]

Appendix S2: Definitions used for maternal morbidity indicators

The proposed EURO-PERISTAT indicator includes both management-based and disease-specific criteria. It is defined as the number of women experiencing any combination of the following conditions or procedures, as a proportion of all women with live and stillborn babies.

-eclamptic seizures

-embolism

-surgery other than caesarean section or tubal ligation

-embolisation of the uterine arteries

-blood transfusion with several possible categories : 3 units of red blood cells or more, 5 units or more, other amount or no units specified.
